# Structure and gating of kainate receptors

**DOI:** 10.3389/fphar.2025.1662316

**Published:** 2025-10-20

**Authors:** Shanti P. Gangwar, Maria V. Yelshanskaya, Laura Y. Yen, Thomas P. Newton, Alexander I. Sobolevsky

**Affiliations:** ^1^ Department of Biochemistry and Molecular Biophysics, Columbia University, New York, NY, United States; ^2^ Cellular and Molecular Physiology and Biophysics Graduate Program, Columbia University Irving Medical Center, New York, NY, United States; ^3^ Cellular, Molecular, and Biomedical Studies Umbrella Program, Columbia University Irving Medical Center, New York, NY, United States

**Keywords:** kainate receptor, cryo-EM, gating mechanism, channel blockers, allosteric modulators, Neto1/2

## Abstract

Ionotropic glutamate receptors (iGluRs) are crucial for fast excitatory neurotransmission in the mammalian central nervous system (CNS). Kainate receptors (KARs), a subclass of iGluRs, are tetrameric, ligand-gated ion channels that play key modulatory roles at both pre- and post-synaptic sites within neuronal circuits and in the regulation of synaptic plasticity. KARs are composed of GluK1-GluK5 subunits, with subunits GluK1-GluK3 forming functional homo- and heteromeric channels, while GluK4-GluK5 assemble as obligate heteromers, partnering with subunits GluK1-GluK3. Over the past two decades, numerous homomeric and heteromeric structures of isolated domains and the full-length receptor have been solved in various functional states, providing detailed descriptions of functional mechanisms, thereby addressing several longstanding questions in the field of KAR biology. These studies revealed overall structural similarity of KARs with other iGluRs, particularly AMPA receptors in the closed and activated states, and the agonist-bound non-conducting state adopting a conformation which is different from other iGluRs. This review highlights recent structural insights into gating and pharmacological regulation of KARs, offering deeper understanding of their roles in synaptic transmission and neuronal signaling.

## 1 Introduction

**FIGURE 1:**
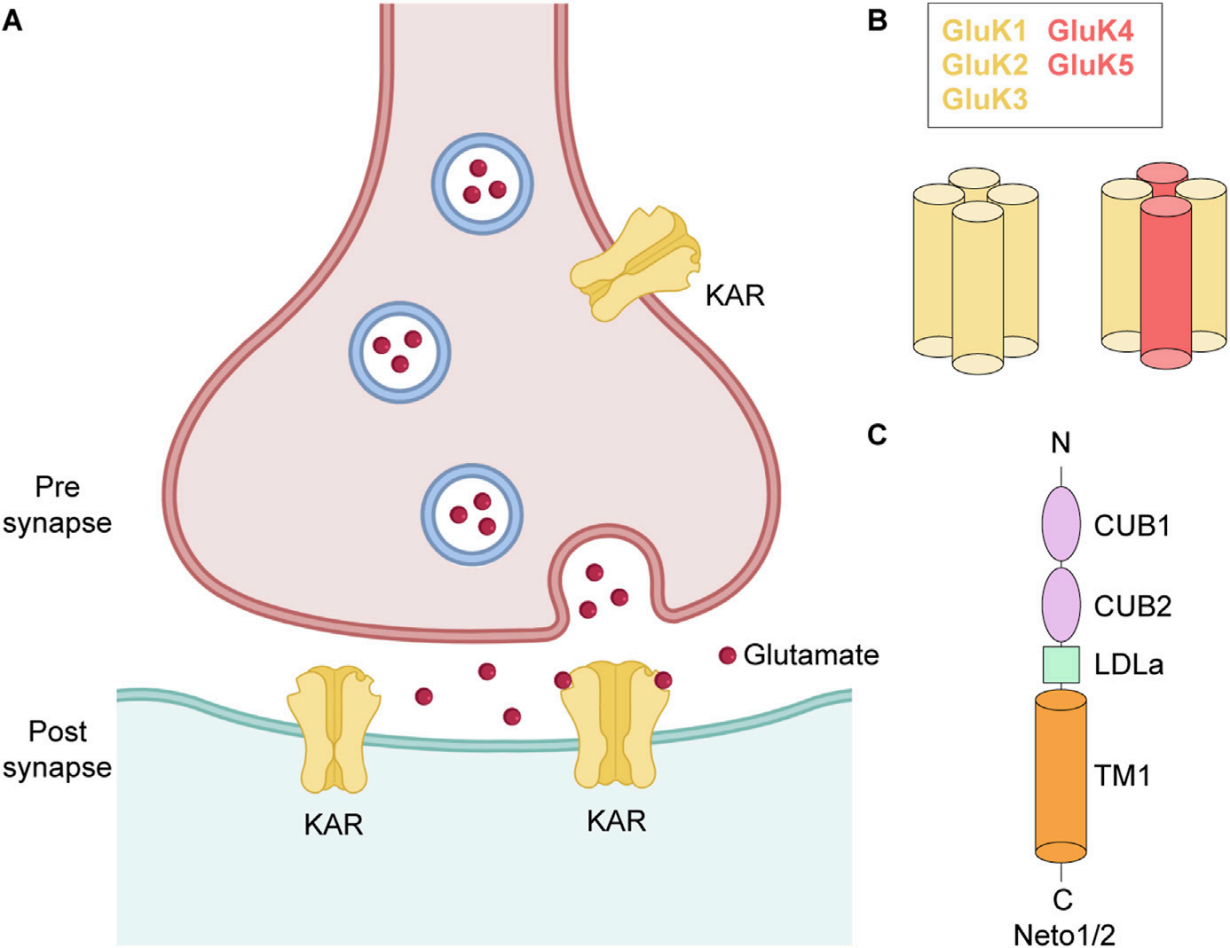
Glutamatergic synapse. **(A)** Pre- and post-synaptic KARs. **(B)** Subunit composition and tetrameric assembly of homomeric and heteromeric KARs. **(C)** Topology of Neto1/2.

Synaptic transmission is a fundamental process within the central nervous system (CNS), relying on receptors that detect neurotransmitter released into the synaptic cleft, initiating a precise and coordinated cellular response. The overall functionality of the CNS is regulated by a balanced interplay between excitatory and inhibitory activity, which is mediated by neurotransmitters glutamate (Glu) and γ-aminobutyric acid (GABA), respectively. Glu serves as the principal excitatory neurotransmitter, playing an essential role in numerous neuronal and cognitive functions, including learning, memory, and synaptic plasticity (Watkins and Evans, 1981; Willard and Koochekpour, 2013; Hansen et al., 2021). The effects of Glu are mediated by ionotropic glutamate receptors (iGluRs), ligand-gated ion channels which are categorized into four distinct subclasses based on their selective agonists: α-amino-3-hydroxy-5-methyl-4-isoxazolepropionic acid receptors (AMPARs), kainate receptors (KARs), *N*-methyl-D-aspartic acid receptors (NMDARs), and δ receptors (GluDs) (Hansen et al., 2021) (Figure 1A). Glu binds AMPARs, KARs and NMDARs, inducing ion channel opening and allowing influx of cations like Na^+^, K^+^, and Ca^2+^ across the membrane into the postsynaptic neuron. This process leads to depolarization of the postsynaptic membrane, triggering subsequent downstream electrical excitation of the post-synaptic neuron (Traynelis et al., 2010; Hansen et al., 2021). In the past four decades, AMPARs and NMDARs have been the primary focus of research due to their role in mediating the majority of fast excitatory neurotransmission and memory formation. KARs, however, have received less attention, despite their significant role in neuronal development, synaptic depolarization, intrinsic cellular excitability, and homeostatic synaptic plasticity gatekeeping (Valbuena and Lerma, 2021). Here, we review recent advances in KAR research.

The KAR family of iGluRs comprises five subunits, GluK1-GluK5, which are encoded by the *GRIK1*-*GRIK5* genes. KARs are widely expressed throughout CNS, with subtype- and region-specific distribution patterns that contribute to their distinct roles in excitatory neurotransmission, synaptic plasticity, and circuit modulation (Lerma, 2006; Vincent and Mulle, 2009). However, the expression profiles of the different subunits are quite heterogenous (Herb et al., 1992; Wisden and Seeburg, 1993; Bahn et al., 1994), dependent on cell type, synaptic localization, and developmental stage (Paternain et al., 2000; Vesikansa et al., 2007). For example, GluK2 and GluK5 are expressed in the majority of hippocampal neurons, as well as in various types of interneurons (Paternain et al., 2000), GluK1 exhibits widespread expression in cerebellar cortex and olfactory bulbs during early development (Dhingra et al., 2024), GluK3 is predominantly expressed in granule cells of the dentate gyrus (DG) (Pinheiro et al., 2007), and GluK4 is mainly present in the pyramidal neurons of the DG and Cornu Ammonis 3 (CA3) region of hippocampus and in the amygdala (Arora et al., 2018). Importantly, these subunit-specific expression profiles also underlie the differential involvement of KARs in neurological and psychiatric disorders, with GluK1 and GluK2 strongly implicated in epilepsy and chronic pain, while GluK4 and GluK5 linked to cognitive impairment, mood dysfunction, and neurodegenerative processes (Lerma and Marques, 2013; Stolz et al., 2021; Negrete-Díaz et al., 2022).

**FIGURE 2:**
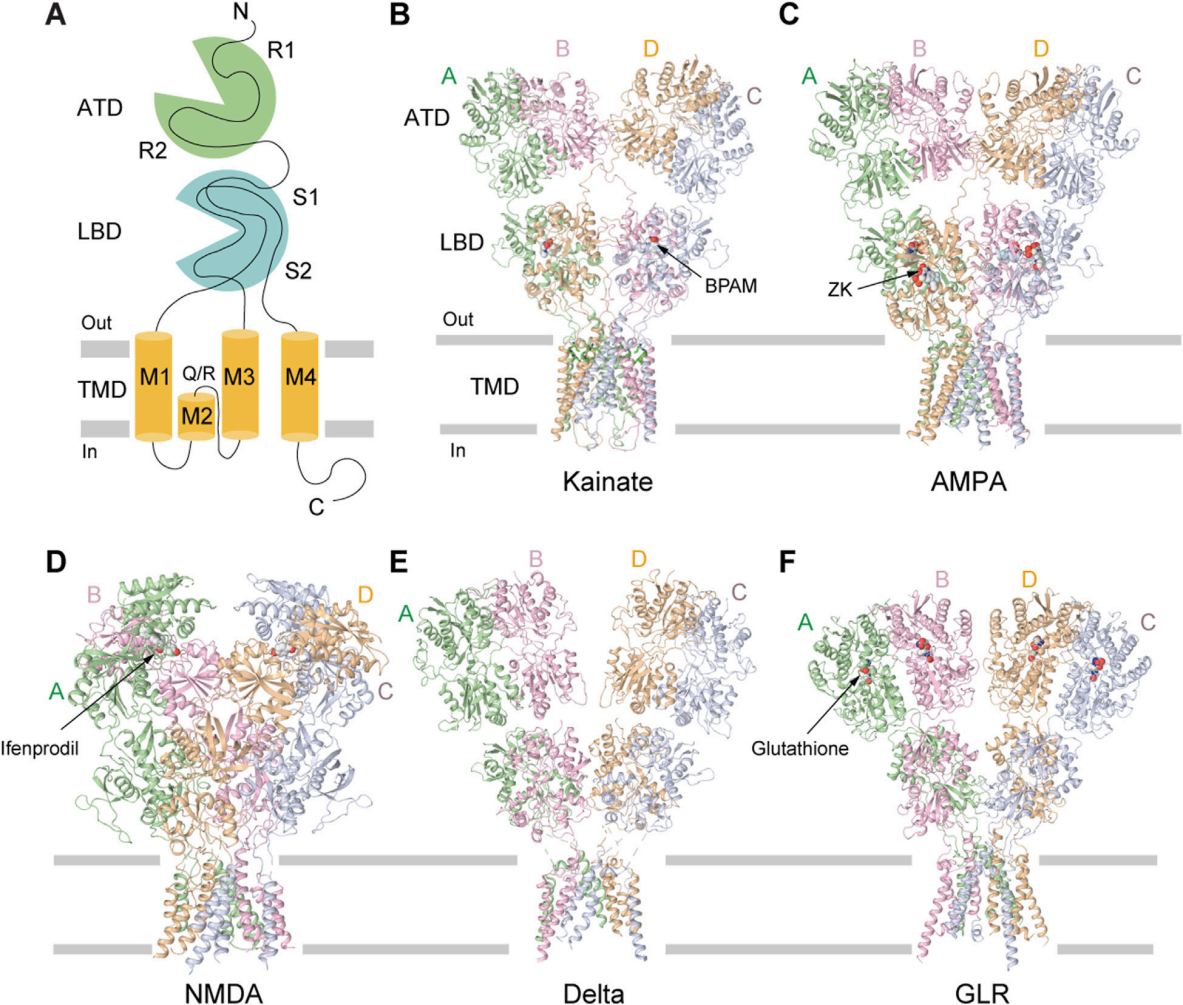
Structures of different iGluR subtypes. **(A)** Membrane topology of iGluR subunit. **(B)** Homomeric KAR GluK2 bound to the positive allosteric modulator BPAM344 (PDB: 8FWQ). **(C)** Homomeric AMPAR GluA2 bound to the competitive antagonist ZK200775 (PDB: 3KG2). **(D)** GluN1/N2B NMDAR bound to the allosteric inhibitor ifenprodil (PDB: 4PE5). **(E)** Homomeric delta receptor GluD1 bound to the competitive antagonist 7-CKA (7-CKA is not resolved, PDB: 6KSS). **(F)** Homomeric plant GLR GLR3.4 bound to glutathione (PDB: 7LZH).

GluK1-GluK3 are low affinity KAR subunits, due to relatively high concentration of agonist needed for their activation (K_D_ for [^3^H]kainate, 50–100 nM) compared to GluK4 and GluK5 which are high affinity subunits (K_D_ for [^3^H]kainate, 5 nM) (Fletcher and Lodge, 1996). The five KAR subunits assemble as either homo- or heterotetrameric complexes (Figure 1B). GluK1-GluK3 subunits can form both homo- and heterotetramers, whereas GluK4 and GluK5 require co-assembly with GluK1-GluK3 subunits to form functional heterotetramers (Egebjerg et al., 1991; Werner et al., 1991; Bettler et al., 1992; Herb et al., 1992). The functional diversity of KARs is further enhanced by alternative splicing of the GluK1 and GluK2 subunits (GluK1a, GluK1b, GluK1c, GluK2a and GluK2b), which produce different isoforms of varying length either at N- or C-termini (Lerma et al., 2001; Jaskolski et al., 2004). These splice variants show altered pharmacological and gating properties like sensitivity to agonists, interaction with auxiliary proteins, synaptic localization and thus fine tune synaptic physiology (Lerma, 2006; Dhingra et al., 2024). Editing of mRNA at the Q/R site, which substitutes an arginine (R) in place of the encoded glutamine (Q), in the re-entrant M2 loop of GluK1 and GluK2 subunits further increases molecular diversity of KARs (Q621 in human and rat GluK2) (Figure 2A). Although the editing at the Q/R site does not impact KAR assembly, it renders GluK1- and GluK2-containing receptors Ca^2+^-impermeable and alters channel conductance (Wilding et al., 2005).

In addition to the core subunits that assemble KAR tetramers, native receptors bind to single-pass transmembrane auxiliary subunits, neuropillin and tolloid-like proteins 1/2 (Neto1/2), which modulate their kinetics, polyamine block, enhance biogenesis, trafficking, and promote targeting to the plasma membrane (Zhang et al., 2009; Straub et al., 2011b; Tomita and Castillo, 2012; Vernon and Swanson, 2017) (Figures 1A,C). While recombinant KARs comprised of GluK1 or GluK2 subunits exhibit similar functional properties, incorporation of Neto1 or Neto2 auxiliary subunits confers significant functional divergence between these receptor types. The modulation of KAR gating and pharmacological properties by Neto proteins appears to be specific to both the receptor subunit composition and the isoform of bound Neto protein (Fisher, 2015). For instance, Neto subunits significantly increase affinity of GluK1 receptors to Glu (∼10–30 times), producing much weaker effect on affinity of GluK2 receptors (∼3-fold increase) (Fisher and Mott, 2012; Li et al., 2019; Palacios-Filardo et al., 2016). At non-saturating concentrations of Glu, both Neto1 and Neto2 slow the rate of the GluK1 homomer desensitization, while only Neto2 produces a comparable deceleration at saturating Glu concentrations (Fisher, 2015). Interestingly, the extent of deceleration is greater for Neto2 compared to Neto1 as well as for GluK1 compared to GluK2 receptors. On the other hand, Neto1 accelerates recovery from desensitization for both GluK1 and GluK2 receptors, whereas Neto2 speeds it up only for GluK2 receptors. Further, co-expression of GluK2 with Neto2 enhances the current amplitude and increases channel open probability (Zhang et al., 2009; Copits and Swanson, 2012; Yan and Tomita, 2012).

Similarly, Neto proteins have been shown to regulate properties of heteromeric KARs. Co-expression of Neto1 with GluK2/GluK5 receptors nearly abolished desensitization at micromolar concentrations of Glu without altering *EC*_50_ for Glu (Fisher and Mott, 2012). In addition, for this combination of subunits, the agonist removal elicited a pronounced rebound current (Fisher and Mott, 2012). Furthermore, Neto1 accelerated the recovery from desensitization of heteromeric GluK2/GluK5 receptors to a comparable extent as for homomeric GluK2 receptors. Co-expression of Neto1 with GluK1/GluK5, GluK3/GluK5, or GluK2/GluK4 accelerated the recovery from desensitization to similar extents, indicating that such regulation by Neto1 is not strongly subunit-dependent (Fisher and Mott, 2012). In contrast, Neto2 slowed desensitization of GluK1, GluK1/GluK5, and GluK2/GluK5 receptors. Moreover, Neto2 increased the rate of recovery from desensitization only for GluK1/GluK5 and GluK2/GluK5 heteromeric KAR subunit assemblies (Straub et al., 2011a; Straub et al., 2011b; Griffith and Swanson, 2015). Collectively, these findings indicate that incorporation of Neto auxiliary subunits into KARs can substantially reshape the kinetic properties of neuronal responses to glutamatergic input (Straub et al., 2011b; Straub et al., 2011a; Copits and Swanson, 2012; Fisher, 2015; Sheng et al., 2015).

Extensive structural investigations utilizing X-ray crystallography have detailed the architecture of individual KAR domains of various subtypes, including the amino-terminal domain (ATD) and ligand binding domain (LBD). These studies have laid the groundwork for understanding the domain architecture of KARs. In recent years, full-length structures of KARs have been elucidated using cryo-electron microscopy (cryo-EM), which has emerged as the principal technique for resolving larger membrane protein complexes at near atomic resolution. In this review, we discuss the recent advancements and findings in structural and functional characterization of homomeric and heteromeric KAR complexes and provide detailed analysis of their molecular architecture, composition, and organization. Furthermore, we describe the mechanisms underlying channel gating, tuning of the receptor activity by positive and negative allosteric modulators and inhibition by polyamine channel blockers. We aim to offer a comprehensive view of the molecular processes underlying KAR function to better understand their roles as key receptors in the CNS.

## 2 Overall structure of KAR

Initial structural characterizations of full-length AMPARs and NMDARs were done by X-ray crystallography (Sobolevsky et al., 2009; Karakas and Furukawa, 2014; Lee et al., 2014). However, attempts to crystallize full-length KARs were unsuccessful. With the advancements in transmission electron microscopy, full-length KAR structures have been solved using single-particle cryo-EM. The first glimpse of the full-length KAR structure was a relatively low-resolution, 7.6-Å reconstruction of homomeric GluK2 in the high-affinity agonist (2S, 4R)-4-methylglutamate (SYM 2081)-bound non-conducting state (Meyerson et al., 2014), which was followed by a similar successor structure with improved 3.8-Å resolution (Meyerson et al., 2016). Since then, numerous structures of full-length homo- and heteromeric KARs in various functional states have been determined. These include homomeric GluK1 bound to agonist Glu (Selvakumar et al., 2021), homomeric GluK2 bound to the positive allosteric modulator (PAM) BPAM344 (BPAM) (Gangwar et al., 2023b), negative allosteric modulator (NAM) perampanel (PMP) (Gangwar et al., 2023b), and ion channel blockers (Gangwar et al., 2024a), homomeric GluK2 in complex with Neto2 bound to the agonist kainic acid (KA) and competitive antagonist 6,7-dinitroquinoxaline-2,3-dione (DNQX) (He et al., 2021), heteromeric GluK2/GluK5 bound to agonist Glu and competitive antagonist 6-cyano-7-nitroquinoxaline-2,3-dione (CNQX) (Khanra et al., 2021), and homomeric GluK3 bound to the agonist SYM 2081 and competitive antagonists UBP301 and UBP310 (Kumari et al., 2019; Kumari et al., 2020). Furthermore, studies utilizing time-resolved cryo-EM have elucidated the activated state of homomeric GluK2 in complex with Glu and PAMs BPAM and plant lectin Concanavalin A (ConA) (Gangwar et al., 2024b). Recent structures of GluK2 shed light on the multi-step process of KAR desensitization (Gangwar et al., 2024b; Zhou et al., 2025) and regulation by partial agonist domoate (Segura-Covarrubias et al., 2025; Zhou et al., 2025).

Structural and compositional diversity in the kainate receptor family has been studied using single-cell RNA sequencing and single-molecule fluorescence (Selvakumar et al., 2021). The study demonstrated that KARs can form di-heteromers with both balanced (2:2) and unbalanced (3:1 and 1:3) stoichiometries and further proposed the potential existence of tri- and tetra-heteromeric assemblies. Specifically, it was reported that GluK1/GluK2, GluK2/GluK3, and GluK1/GluK3 combinations can generate di-heteromers in all balanced or unbalanced stoichiometries, with their distributions determined by the relative expression levels of each subunit. Prior assumptions regarding the GluK5 assembly rules were revised (Kumar et al., 2011; Reiner et al., 2012) by illustrating the existence of KAR subunit complexes containing a single GluK5 subunit (Selvakumar et al., 2021). Although GluK4 was not examined, it was hypothesized to exhibit analogous assembly properties to those of GluK5. Collectively, these findings indicate that KARs can form tri- and tetra-heteromeric receptors, underscoring the level of KAR structural and functional diversity comparable to AMPA and NMDA receptors.

The overall architecture of tetrameric KARs follows a canonical “dimer-of-dimers” iGluR subunit arrangement, exhibiting an approximate overall two-fold rotational symmetry and Y-shaped, three-layer domain organization (Meyerson et al., 2016; Kumari et al., 2020; He et al., 2021; Khanra et al., 2021; Gangwar et al., 2023b) (Figures 2A–F). The uppermost layer comprises four amino-terminal domains (ATDs), each connected by a linker to the corresponding ligand-binding domain (LBD) in the intermediate layer (Meyerson et al., 2014; Gangwar et al., 2023b). At the base of the structure is a cation-selective ion channel, formed by the transmembrane domains (TMDs) of four subunits, each connected to the corresponding LBD by three linkers, S1-M1, M3-S2, and S2-M4 (Meyerson et al., 2014; Gangwar et al., 2023b) (Figure 2A). Similar to AMPARs, the tetrameric structure of KARs features two conformationally distinct but chemically identical diagonal subunit pairs (A/C and B/D), with domain swapping occurring between the ATD and LBD layers (Meyerson et al., 2016; Gangwar et al., 2023b; Gangwar et al., 2024b). KAR assembly is primarily orchestrated by the ATDs, which form two distinct dimers (A/B and C/D) (Das et al., 2010; Kumar et al., 2011; Zhao et al., 2017). Domain swapping between ATD and LBD layers leads to formation of LBD dimers A/D and B/C. Heteromeric GluK2/GluK5 receptors assemble from two copies of individual subunits, which organize in a 2:2 stoichiometry, with GluK2 occupying the B/D positions and GluK5 the A/C positions (Khanra et al., 2021). Consequently, in the resulting heterotetramer, both the ATD and LBD layers are organized as dimers of two heterodimers. Although heteromeric GluK2/GluK5 are the most abundant KARs in the brain, homomeric GluK2 receptors have been the primary focus of structural and functional studies because of their excellent biochemical behavior (Porter et al., 1997).

### 2.1 Amino terminal domain (ATD)

**FIGURE 3:**
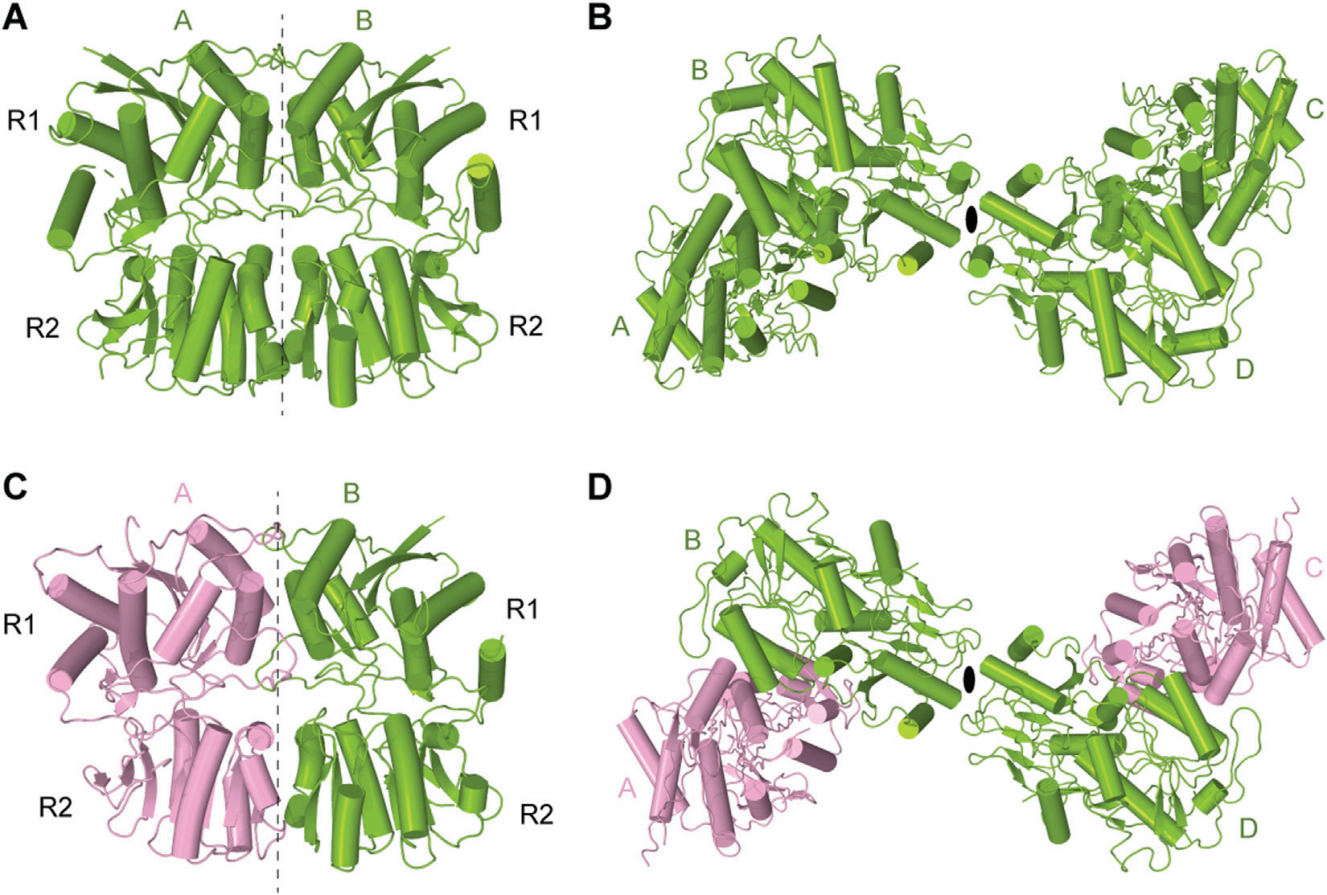
Dimers and tetramers of KAR amino-terminal domain (ATD). **(A)** Crystal structure of ATD dimer of GluK2 (PDB: 3H6G). **(B)** Cryo-EM structure of tetrameric ATD of GluK2 (PDB: 8FWT). **(C)** Dimeric assembly of GluK2/GluK5 ATD (PDB: 3QLU). GluK2 is shown in green and GluK5 in pink. **(D)** Tetrameric assembly of GluK2/GluK5 ATD (PDB: 3QLV).

The amino-terminal domain (ATD) of KARs plays a pivotal role in shaping the overall receptor architecture by directing their assembly and determining subunit arrangement within the tetrameric complex (Das et al., 2010; Kumar et al., 2011). This domain is essential for the initial dimerization of subunits, a critical step in the formation of functional tetramers (Figures 3A–D) (Das et al., 2010). Structurally, each ATD has a clamshell-like structure made of the upper R1 and lower R2 lobes (Figures 3A,C). Two individual ATDs assemble into ATD dimers (Figures 3A,C), while two ATD dimers assemble into a dimer-of-dimers configuration in the context of the KAR tetramer (Kumar et al., 2009; Kumar et al., 2011; Kumar and Mayer, 2010) (Figures 3B,D). The ATDs form homo- or heterodimers, with specific molecular pairing influencing the final subunit composition and spatial organization of the receptor (Kumar et al., 2009; Kumar et al., 2011; Kumar and Mayer, 2010). For example, in heteromeric GluK2/K5, the ATDs organize into pairs of GluK2/K5 heterodimers. In this configuration, GluK5 subunits occupy peripheral A/C positions within the tetramer, while GluK2 subunits are centrally located at positions B/D, facilitating the dimer-of-dimers assembly along the receptor’s two-fold axis of symmetry (Kumar et al., 2011; Khanra et al., 2021) (Figures 3C,D). The energetics of dimerization and subsequent tetramerization favor specific subunit combinations, contributing to the selective formation and stabilization of receptor subtypes *in vivo*. For instance, dimerization of GluK2/K5 heterodimers (K_D_ = 77 nM) occurs much more robustly and favorably compared to GluK2 homodimers (K_D_ = 350 nM), contributing to the greater abundance of GluK2/K5 heterotetramers in the brain when compared to GluK2 homotetramers (Chaudhry et al., 2009; Kumar et al., 2011). Moreover, the ATD has been implicated in receptor trafficking, underscoring its integral role in KAR structural maturation, functional modulation, and regulation of synaptic architecture and ultrastructure (Zhao et al., 2017). Until now, no modulation of AMPARs or KARs through binding of small molecules to the ATD has been described. This is in sharp contrast to NMDARs (Zhu and Paoletti, 2015) and plant glutamate receptor-like channels (GLRs) (Green et al., 2021), which can be functionally regulated by allosteric modulators that bind to ATDs. For instance, ATD-bound ifenprodil, Zn^2+^, and polyamines modulate the activity of NMDARs (Zhou and Tajima, 2023), while glutathione regulates function of GLRs (Li et al., 2013; Green et al., 2021) (Figures 2D,F). However, no evidence is available on whether glutathione or ifenprodil bind to the ATD in KARs or modulate KAR function.

### 2.2 Ligand binding domain (LBD)

**FIGURE 4:**
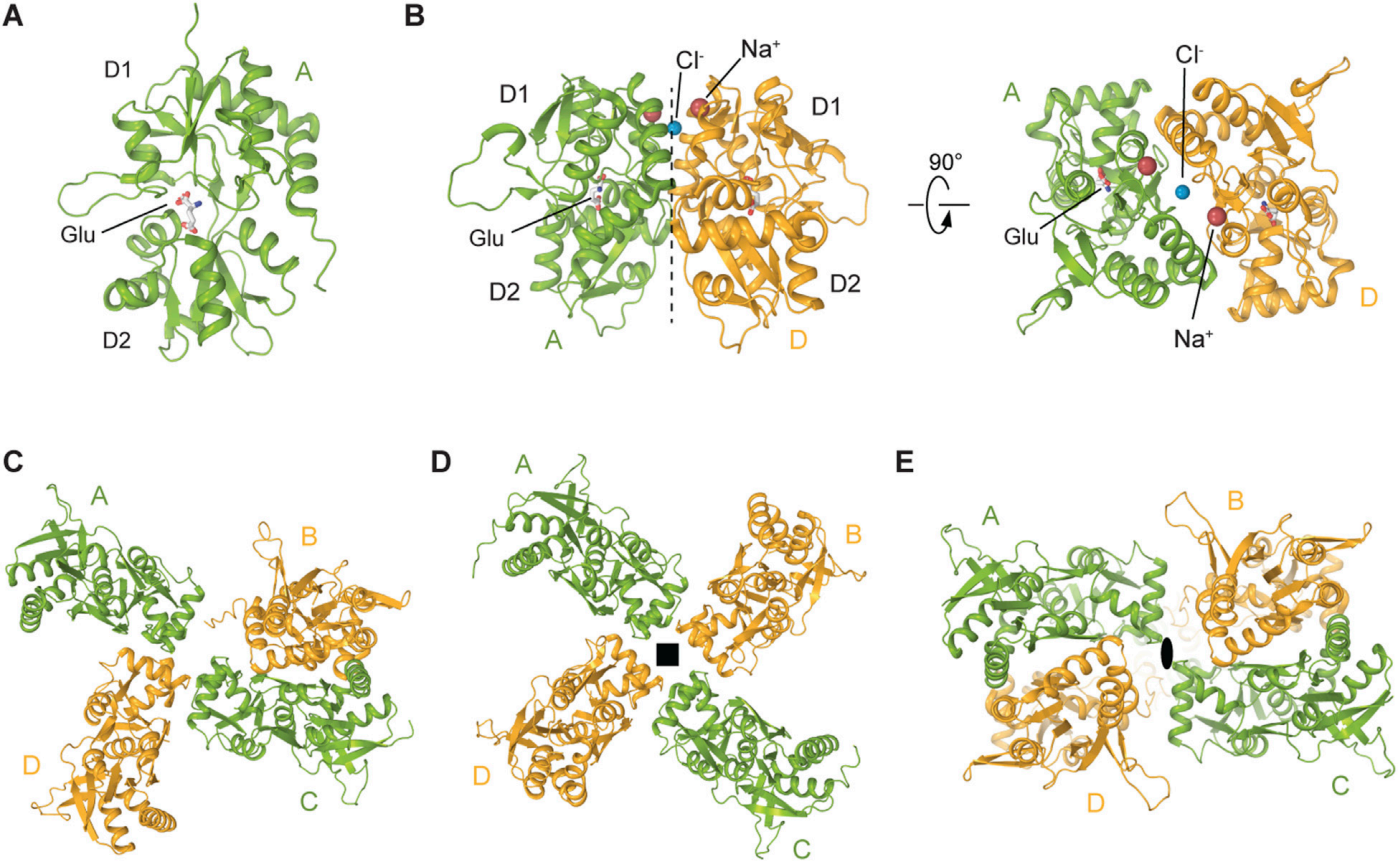
Structures and assemblies of ligand binding domains (LBDs) in KARs. **(A)** Crystal structure of GluK2 LBD bound to L-Glu (PDB: 1S50). **(B)** Back-to-back GluK2 LBD dimer (PDB: 2XXR). Sodium ion at the apex of D1-D1 interface is shown as a red sphere and chloride ion as a blue sphere. **(C)** Cryo-EM structure of the asymmetric GluK2 tetrameric LBD layer in the putative apo state (PDB: 9C5Z). **(D)** Cryo-EM structure of the four-fold GluK2 tetrameric LBD layer representing another putative apo state (PDB: 8F0O). **(E)** Cryo-EM structure of the GluK2 dimer-of-dimers tetrameric LBD layer (PDB: 8FWS). Black square and oval in D and E indicate four-fold and two-fold rotational symmetry, respectively.

Over the past two decades, several crystal structures of ligand binding domains (LBD) from homomeric or heteromeric KARs in complex with various agonists and competitive antagonists have been reported (Mayer, 2005; Mayer et al., 2006; Hald et al., 2007; Nayeem et al., 2011; Venskutonyte et al., 2011; Venskutonytė et al., 2012; Veran et al., 2012; Assaf et al., 2013; Kristensen et al., 2016; Kumari et al., 2019). These structures showed that each LBD is composed of two polypeptide stretches, S1 and S2, which are separated by the membrane segments M1-M3 (Figure 2A). S1 and S2 assemble a clamshell-shape structure of the LBD, with an upper lobe D1 and lower lobe D2 (Figure 4A). The binding site for agonists and competitive antagonists is located within the clamshell, in the cleft between D1 and D2 (Mayer, 2005; Mayer et al., 2006; Chaudhry et al., 2009; Larsen et al., 2017; Bay et al., 2024b; 2024c; 2024a). The structures of isolated LBDs reveal the back-to-back dimeric arrangement of individual protomers (Figure 4B), with PAMs binding at the LBD dimer interface, between two protomers (Figures 4B, 7C) (Mayer, 2005; Larsen et al., 2017; Bay et al., 2024b). Crystallographic analyses of isolated KAR LBDs have identified binding sites for Na^+^ and Cl^−^ ions at the LBD dimer interface, demonstrating that ion binding modulates receptor desensitization by constraining rearrangements of the dimer interface (Nayeem et al., 2009; 2011; Dawe et al., 2013). For example, for the GluK2 D776K mutant, the positive charge of the lysine substitutes the positively charged Na^+^ ion, enhancing GluK2 activation and inhibits desensitization (Plested et al., 2008; Chaudhry et al., 2009; Nayeem et al., 2009; Nayeem et al., 2011; Dawe et al., 2013) (Figure 4B).

The full-length cryo-EM structures of KARs revealed three major arrangements of protomers in the LBD layer: the canonical dimer-of-dimers (AMPAR-like), an atypical asymmetric, and a pseudo four-fold symmetrical configurations (Meyerson et al., 2016; Kumari et al., 2019; Gangwar et al., 2023b; Gangwar et al., 2024b; Zhou et al., 2025) (Figures 4C–E). The pseudo four-fold symmetrical arrangement of LBDs (Figure 4D) was first observed in the SYM2081-bound structure of GluK2 with the apparently closed ion channel pore and was proposed to represent a desensitized state (Meyerson et al., 2016). Making a parallel with AMPARs, where desensitization is accompanied by rupture of the D1-D1 interface in LBD dimers (Twomey et al., 2017b; Klykov et al., 2021), this pseudo four-fold symmetrical LBD layer, an exaggerated form of two LBD dimers with fully ruptured interfaces, is consistent with the recovery from desensitization in KARs being substantially slower compared to AMPARs (Weston et al., 2006).

### 2.3 Transmembrane domain (TMD)

iGluRs are members of the pore-loop superfamily of tetrameric ion channels, sharing structural homology with potassium channels, voltage-gated sodium and calcium channels, cyclic nucleotide-gated (CNG) channels, and transient receptor potential (TRP) channels. The key characteristic of this superfamily is a pore domain comprised of two transmembrane segments connected by a re-entrant pore loop, which enters and exits the membrane on the same side and typically serves as a selectivity filter (Wollmuth and Sobolevsky, 2004; Huettner, 2015; Tikhonov and Zhorov, 2020). In voltage-gated, CNG, and TRP channels, the pore domain is formed by the transmembrane helices S5 and S6 and a re-entrant pore loop (P-loop) between them. Similarly, the pore domain in iGluRs is formed by the M1 and M3 transmembrane helices with a re-entrant M2 loop between them (Sobolevsky et al., 2009; Stroebel and Paoletti, 2021), but the entire construction is flipped upside-down within the plasma membrane compared to voltage-gated, CNG and TRP channels. The M3 helices cross near their extracellular apex to form an activation gate, which opens by outward splaying, a mechanism conserved across AMPA, kainate, NMDA, and likely GluD receptors (Chen et al., 2017; Twomey et al., 2017a; Chou et al., 2024; Gangwar et al., 2024b). A conserved motif within M3, SYTANLAAF, is critical for gating and undergoes conformational changes that permit pore opening. The M2 loop creates a narrow intracellular constriction that governs ion selectivity, Ca^2+^ permeability, conductance, polyamine block and is crucial in determining inward rectification, while M4 may contribute to tetramer stabilization (Wollmuth and Sobolevsky, 2004; Sobolevsky et al., 2009; Karakas and Furukawa, 2014; Meyerson et al., 2014; Huettner, 2015).

**FIGURE 5:**
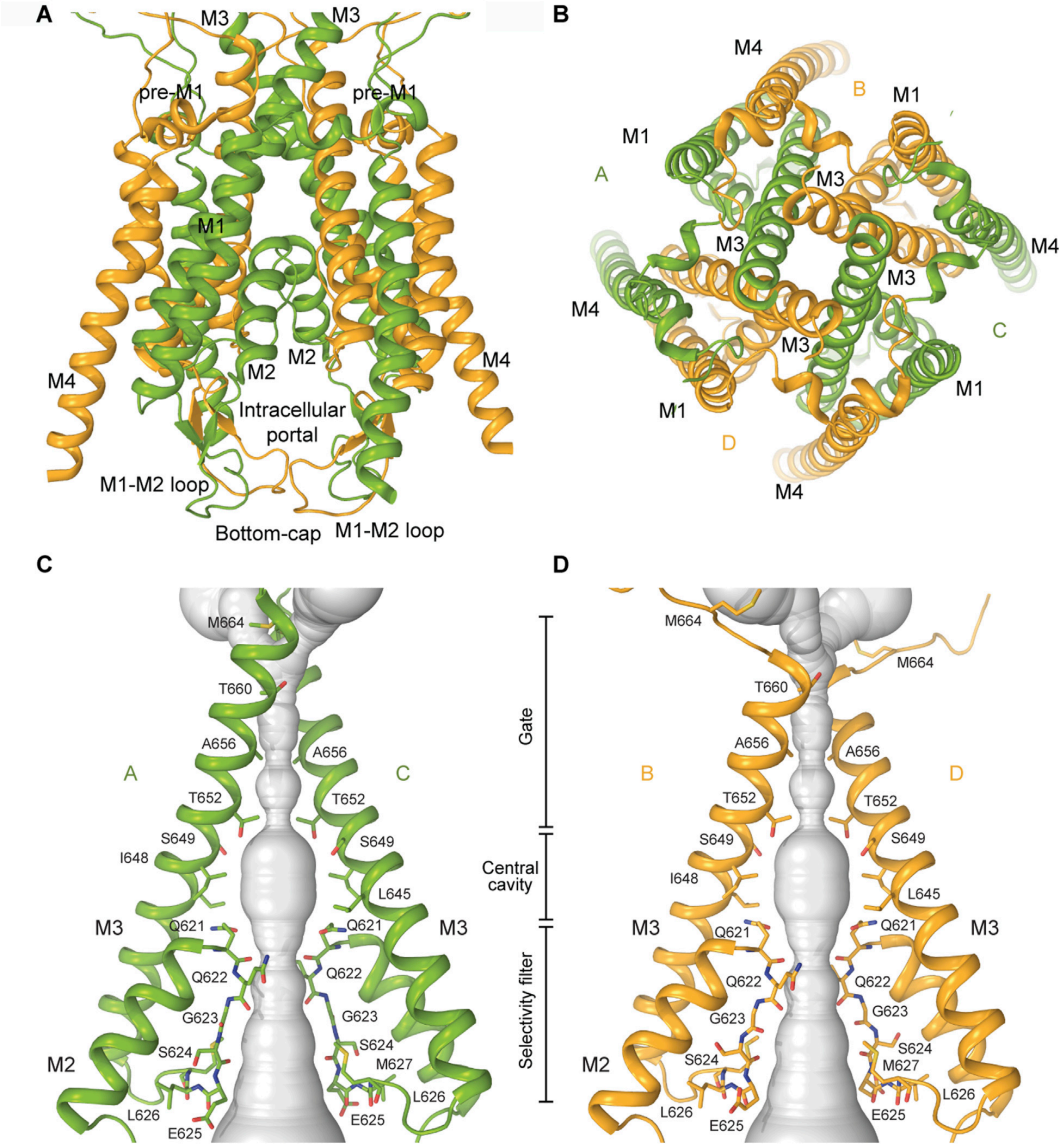
Transmembrane domain of KARs. **(A)** TMD of GluK2 in the closed state with all four transmembrane helices M1-M4 shown (PDB: 8FWS). The M1-M2 loops forming a bottom cap are shown at the bottom of the TMD. **(B)** TMD viewed extracellularly. **(C,D)** Pore-forming segments M2 and M3 in subunits A and C **(C)** or B and D **(D)** of the closed-state GluK2 structure, with residues lining the pore shown as sticks. The pore profile is shown as a space-filling model (grey).

In KARs, the TMD consists of four segments (M1–M4), with M2 and M3 lining the pore, and M1 and M4 serving as peripheral helices that provide stability to the tetrameric core (Gan et al., 2015) (Figures 2A, 5A,B). The M3 helices line the extracellular portion of the ion channel pore and form the upper gate, whereas the M2 loops line the intracellular segment of the pore and form the selectivity filter (Sobolevsky et al., 2009; Karakas and Furukawa, 2014; Lee et al., 2014; Meyerson et al., 2016; Gangwar et al., 2023b). Structures of KARs and other iGluRs reveal that the tips of the M2 loops of all four subunits converge at the channel’s narrowest region, located approximately midway across the membrane (Figure 5A). The M2 loop creates a constricted segment within the pore, which governs ion selectivity, permeation, and channel block, possibly playing the role of a lower gate in the channel (Wollmuth and Sobolevsky, 2004; Traynelis et al., 2010; Twomey et al., 2017a; Gangwar et al., 2024a). The intracellular segment of M4 is directly linked to the highly flexible and disordered C-terminal domain (CTD), which influences receptor gating, but has not yet been structurally resolved in any iGluRs. In addition to the transmembrane helices, the M3-S2 LBD-TMD linkers undergo significant conformational changes and participate in KAR channel gating (Gangwar et al., 2024b).

## 3 Structural determinants of Neto2 binding to KARs

**FIGURE 6:**
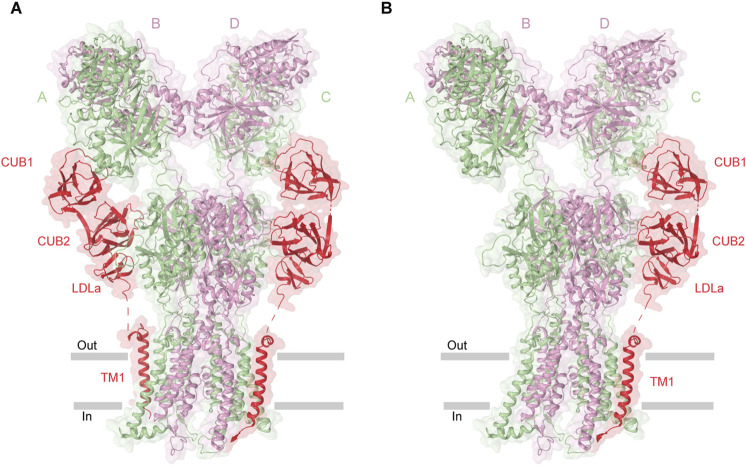
Structure of GluK2-Neto2 complex. **(A)** Homomeric GluK2 in complex with two Neto2 molecules (PDB: 9N4Q). **(B)** GluK2 bound to one Neto2 molecule (PDB: 9N4P). Neto2, shown in red, shares interfaces with the receptor.

Native KARs assemble into macromolecular complexes with the auxiliary subunits Neto1 and Neto2. Both Neto1 and Neto2 exhibit an identical domain architecture, characterized by the presence of two CUB (complement C1r/C1s, Uegf, Bmp1) domains and one LDLa (low-density lipoprotein receptor class A) domain (Figure 1C) (Lerma, 2011; Yan and Tomita, 2012). While structures of KAR-Neto1 complex remain unresolved, recent structural studies of the GluK2-Neto2 complex in different states resolved all four principal domains of Neto2, namely CUB1, CUB2, LDLa, and the C-terminal transmembrane domain, which are arranged consecutively along the receptor’s surface following the extracellular to intracellular direction (He et al., 2021; Gangwar et al., 2025). The structures revealed that Neto2 can bind to the receptor with varying stoichiometry, either as one or two Neto2 protomers per GluK2 tetramer. However, the functional impact of Neto2 on the receptor appears to be stoichiometry independent (He et al., 2021; Gangwar et al., 2025) (Figures 6A,B). In the GluK2-Neto2 complex, the extracellular portion of Neto2 establishes extensive interactions with the ATD and LBD layers of the receptor. Specifically, the CUB1 domain interacts with the A and C subunits of GluK2, while the CUB2 domain engages with Loop 1 in the upper D1 lobe of the LBDs of subunits B and D. The LDLa domain links the protein to the lower D2 lobe of the GluK2 LBD within the neighboring LBD dimer. Specifically, the CUB2 and LDLa domains interact with LBDs from GluK2 subunits A/B (or C/D) (Figures 6A,B). Consequently, the CUB2 and LDLa domains of Neto2 mediate cross-interactions with protomers from two distinct LBD dimers, positioning them to regulate the relative orientation of these dimers during receptor gating. The interface between GluK2 and Neto2 involves the transmembrane helix M1 of GluK2, which contacts the α1 helix and TM1 of Neto2. Finally, the M1-M2 loop of GluK2, forming the intracellular bottom cap and portals, primarily associates with the C-terminal region of Neto2 (He et al., 2021; Gangwar et al., 2025).

## 4 Gating

### 4.1 Closed resting state

**FIGURE 7:**
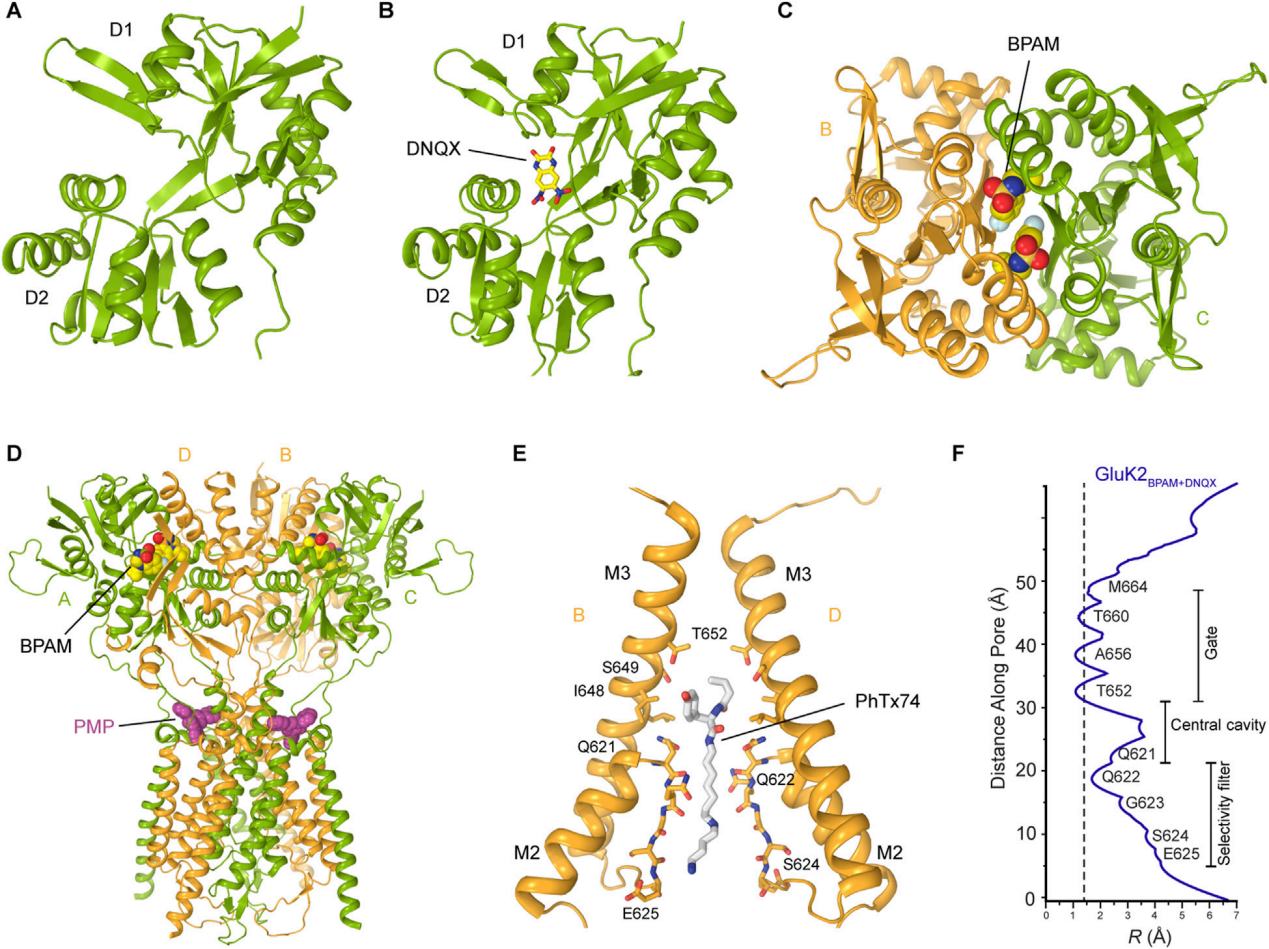
Kainate receptor in the closed state. **(A)** Closed state GluK2 LBD showing the empty open clamshell (PDB: 8FWS). **(B)** GluK2 LBD bound to the competitive antagonist DNQX (PDB: 8FWU). The same site accommodates CNQX, another competitive KAR antagonist, similar to DNQX. **(C)** Two molecules of the positive allosteric modulator BPAM bind symmetrically at the LBD dimer interface (PDB: 8FWS). **(D)** Perampanel binds at the extracellular collar region acting as a wedge (PDB: 8FWW). **(E)** Binding of a representative channel blocker, PhTx-74, in the ion channel pore (PDB: 9DXQ). Only two subunits (B,D) are shown, with the front and back subunits (A,C) omitted for clarity. NpTx-8, Kukomaine A, and spermine bind at the same site in the pore region. **(F)** Pore radius for the closed-state structure of GluK2 bound to BPAM and DNQX. The vertical dashed line denotes the radius of a water molecule, 1.4 Å.

In the closed state, two-fold rotational symmetry is observed between individual ATD dimers and within the ATD dimer-of-dimers arrangement (Figures 3A–D), while the TMD displays a pseudo four-fold symmetry. In contrast, various symmetrical arrangements have been reported in the LBD layer (Figures 4C–E). For example, the LBD layer in antagonist-bound, homomeric GluK3 (Kumari et al., 2019) or the putative apo state homomeric GluK2 (Zhou et al., 2025) was shown to adopt an asymmetric arrangement, with two LBDs forming a dimer and the other two LBDs representing individual monomers (Figure 4C). At the same time, another putative apo state structure of GluK2 obtained in the absence of added ligands exhibited a four-fold symmetrical LBD layer (Figure 4D) (Zhou et al., 2025). Surprisingly, the LBD clamshells in this structure were completely closed, resembling an agonist-bound conformation, suggesting possible presence of endogenous agonists. In contrast, the antagonist-bound heteromeric GluK2/GluK5 and GluK2-Neto2 assemblies adopted the canonical two-fold symmetrical dimer-of-dimers arrangement of LBDs (He et al., 2021; Khanra et al., 2021) (Figure 4E). Intriguingly, the presence of competitive antagonists alone was incapable of stabilizing the homomeric GluK2 or GluK3 LBD layer in the dimer-of-dimers configuration (Kumari et al., 2019; He et al., 2021; Zhou et al., 2025) suggesting that the LBDs of homomeric KARs are more dynamic and prone to conformational instability. When GluK2 is bound to the PAM BPAM, the LBD assumes a single stable conformation, characterized by the strengthened D1–D1 interfaces of LBD dimers as well as the canonical two-fold symmetrical dimer-of-dimers arrangement of LBDs (Gangwar et al., 2023b) (Figure 4E). In this BPAM-stabilized state, the LBD clamshells are completely open and show no traces of bound ligands inside the agonist binding pockets and closed channel pore (Figures 7A–F). Moreover, the BPAM molecules bind symmetrically at the LBD dimer interfaces, positioned around the axis of local two-fold rotational symmetry (Figure 7C).

However, the true KAR apo state, with no ligands bound and the canonical two-fold symmetrical LBD layer, has only been solved very recently (Figures 4E, 7A) (Gangwar et al., 2025). To determine this structure, the purified GluK2 protein was first incubated with BPAM, locking the LBD clamshells in an open conformation and forcing the release of possible contaminating endogenous agonists. The protein was then subjected to an additional chromatographic purification step to ensure BPAM removal, resulting in a protein sample free of ligands (Gangwar et al., 2025). Cryo-EM analysis of the GluK2 receptor and the GluK2-Neto2 complex prepared via this method yielded 3D reconstructions of the true apo state, an open LBD clamshell, with the canonical two-fold symmetrical dimer-of-dimers arrangement of LBDs and no traces of bound ligands (Gangwar et al., 2025).

Interestingly, in the closed antagonist-bound state of the GluK2/GluK5 heterotetramer or GluK3 homotetramer, the M2 loop and the selectivity filter of the channel were unresolved (Kumari et al., 2019; Khanra et al., 2021). In contrast, homomeric GluK2 and GluK2-Neto2 showed well-resolved M2 loops (He et al., 2021; Gangwar et al., 2023b; Gangwar et al., 2024a). Moreover, the latter structures revealed a unique structural feature present in KAR structures but absent in AMPARs and NMDARs: the intracellular cap formed by the M1-M2 loops (Figure 5A). These loops form a dome-like structure beneath the intracellular entrance of the ion channel, acting as a barrier to ions and water moving along the pore axis (He et al., 2021; Gangwar et al., 2023b; Gangwar et al., 2024a). However, the M1-M2 loops form lateral passages that allow permeating ions and water to travel through four intracellular portals running parallel to the membrane, just below the intracellular membrane leaflet. These portals are predominantly lined with polar and charged residues, which facilitate ion and water permeation. In its extracellular gate region, the channel pore is tightly closed by the bundle crossing of the M3 helices (Figures 5B, 7E,F). In all closed-state KAR structures, apo or competitive antagonist-bound, the pore geometry in this region is strikingly similar, with the pore radii smaller than the diameter of a water molecule (He et al., 2021; Khanra et al., 2021; Gangwar et al., 2023b; Gangwar et al., 2024a) (Figure 7F). Similarly, the closed-state structures of GluK2 bound to the non-competitive inhibitor antiepileptic drug perampanel (Gangwar et al., 2023b), or polyamine blockers (Gangwar et al., 2024a), all solved in the presence of BPAM, displayed the same canonical two-fold symmetrical dimer-of-dimers arrangement of LBDs (Figures 7C–F).

#### 4.1.1 Regulation by competitive antagonists

DNQX and CNQX are widely recognized as competitive antagonists of KARs (Chałupnik and Szymańska, 2023). Upon application to neuronal preparations, DNQX and CNQX robustly suppress KAR-mediated synaptic currents, thereby attenuating excitatory neurotransmission facilitated by these receptors (Honoré et al., 1988; Sheardown et al., 1990). Recent cryo-EM studies of GluK2, GluK2/GluK5, and GluK2-Neto complexes revealed that both DNQX and CNQX occupy the agonist-binding site within the LBD of KARs between D1 and D2 lobes (He et al., 2021; Khanra et al., 2021; Gangwar et al., 2023b). This interaction promotes an open conformation of the LBD clamshells, stabilizing the receptor in a non-conducting, closed state (Figure 7B). Thus, by preventing the conformational closure of the LBD essential for channel activation, DNQX and CNQX effectively inhibit receptor function.

#### 4.1.2 Regulation of KAR by perampanel

Perampanel (PMP), initially characterized as a non-competitive inhibitor of AMPARs (Ceolin et al., 2012; Yelshanskaya et al., 2016; Gangwar et al., 2023a), has recently been demonstrated to function as a NAM of KARs, exhibiting concentration-dependent inhibition of KAR activity (Taniguchi et al., 2022; Gangwar et al., 2023b). Structurally, PMP binds to the extracellular collar of the GluK2 KAR channel, which is formed by the pre-M1 region and extracellular segments of the M1, M3, and M4 transmembrane helices, analogous to its binding mode in AMPARs (Figure 7D) (Gangwar et al., 2023b). However, unlike AMPARs, where PMP binding sites are fully occupied across all four subunits, PMP binding in KARs was observed only at two sites associated with subunits A and C, but not B and D (Figure 7D), acting as a wedge, preventing the conformational rearrangements in the TMD region required for channel opening and thereby stabilizing the channel in its closed conformation. Interestingly, PMP exhibits a substantially higher affinity for heteromeric KARs containing the GluK5 subunit, comparable to its affinity for AMPARs, suggesting that such heteromeric KAR assemblies may achieve full occupancy by PMP (Taniguchi et al., 2022). The corresponding structural characterization of heteromeric KAR in complex with PMP remains to be performed.

#### 4.1.3 Regulation of KARs by channel blockers

Positively charged polyamine molecules are ubiquitously present in bacterial, plant, and animal cells and frequently function as blockers of cation-selective ion channels (Tabor and Tabor, 1984). Owing to their cationic nature, polyamines exhibit strong binding affinity to electronegative pores of cation-selective ion channels, often in the micromolar range (Nichols and Lopatin, 1997; Haghighi and Cooper, 1998; Dingledine et al., 1999; Lu and Ding, 1999). As permeant ion channel blockers of AMPARs and KARs, cytoplasmic polyamines (spermine, spermidine, putrescine) have been identified as critical modulators of neuronal signaling, influencing action potential firing rates as well as the efficacy of neurotransmission (Bowie and Mayer, 1995; Nichols and Lopatin, 1997). Recent investigations have elucidated the structural basis underlying the trapping mechanism and inhibition of homomeric GluK2 KAR by a set of synthetic and natural channel blockers, including NpTx-8, PhTx-74, Kukoamine A, and spermine (Gangwar et al., 2024a) (Figure 7E). The trapping mechanism implies that a blocker can enter the open ion channel followed by gate closure behind the blocker, thereby trapping it inside the ion channel pore until the channel is opened again. Structurally, these channel blockers are positioned within the GluK2 ion channel pore, located intracellular to the closed M3 helix bundle-crossing gate. Their hydrophobic moieties occupy the central cavity, while their positively charged polyamine tails extend into the selectivity filter (Figure 7E) (Gangwar et al., 2024a). Although the existing evidence indicates that Neto auxiliary proteins attenuate the channel-blocking effects (Fisher and Mott, 2012; Brown et al., 2016; Bowie, 2018), the precise structural mechanisms underlying this modulation are yet to be elucidated.

### 4.2 Agonist-bound non-conducting states

**FIGURE 8:**
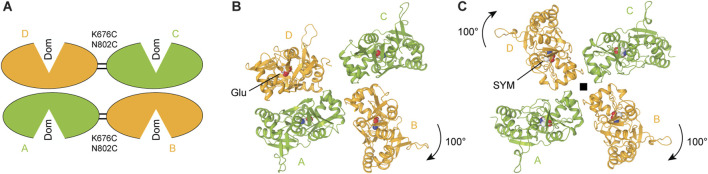
Agonist bound non-conducting states and mechanism of desensitization. **(A)** LBD tetramer in the partial agonist domoate (Dom)-bound and cysteine-stabilized GluK2 with dimer-of-dimers domain arrangement representing a putative shallow desensitized state (schematic based on PDB: 9MZO). **(B)** L-Glu bound GluK2 with asymmetric domain arrangement representing a putative intermediate desensitized state (PDB: 9B39). **(C)** SYM 2081 (SYM)-bound GluK2 with pseudo four-fold symmetrical domain arrangement representing a putative deep desensitized state (PDB: 5KUF).

The first full-length KAR structure in the agonist-bound non-conducting state was a cryo-EM structure of homomeric GluK2 bound to the high-affinity agonist SYM 2081 (Meyerson et al., 2014). This and following structures of agonist-bound KARs revealed the canonical dimer-of-dimers domain arrangement in the ATD layer, similar to AMPARs (Meyerson et al., 2016; Kumari et al., 2019; He et al., 2021; Khanra et al., 2021; Gangwar et al., 2024b; Segura-Covarrubias et al., 2025; Zhou et al., 2025). In contrast, the LBD layer adopts multiple conformations in agonist bound states (Figures 8A–C). The original agonist-bound non-conducting structures of KARs showed a pseudo four-fold symmetrical LBD arrangement (Meyerson et al., 2016; Kumari et al., 2020; Khanra et al., 2021; Gangwar et al., 2024b; Zhou et al., 2025) (Figure 8C). As the state represented by these structures was termed desensitized, desensitization was postulated to involve significant reorganization of the LBD layer without substantial alteration of the ATD and TMD layers. Each individual LBD in these structures is represented by a closed, agonist-bound clamshell, consistent with the isolated agonist-bound LBD structures (Figure 4A). The ion channel in these putative desensitized state structures adopts a closed non-conducting conformation, very similar to the apo resting state (Figure 7F). A comparison of the agonist-bound non-conducting (putative desensitized) and apo closed states of GluK2 reveals the rotation of the B/D subunit LBDs by ∼100° and A/C subunit LBDs by only ∼13°. These rearrangements in the agonist-bound homomeric GluK2 and GluK3 (Meyerson et al., 2016; Kumari et al., 2019) or heteromeric GluK2/GluK5 (Khanra et al., 2021) produce pseudo four-fold symmetric organization of domains in the LBD layer (Figure 8C). This domain organization is characterized by establishment of a ring-like structure termed the ‘desensitization ring’, first postulated for the agonist-bound GluK2 homomer structure (Meyerson et al., 2016). Comparison of the homomeric and heteromeric agonist-bound non-conducting KAR structures showed global similarities in the LBD layers with subtle differences in LBD arrangement in GluK2/GluK5 compared to GluK2, illustrated by LBD dimer-dimer distance of 49.9 Å in GluK2/GluK5 and 54.9 Å in GluK2, suggesting a more compact desensitization ring in the heterotetramer (Khanra et al., 2021). Since GluK5 binds Glu with higher affinity and favors slower deactivation kinetics compared to GluK2, the GluK2/K5 heterotetramer can be activated by the ligand that binds to GluK5 subunits only, without binding to GluK2 subunits (Fisher and Housley, 2013).

Along with the agonist-bound structure characterized by four-fold symmetrical LBD layer, a recent study of GluK2 revealed a Glu-bound structure, where one LBD dimer was preserved, while the other LBD dimer dissociated through ∼100° rotation of one protomer relative to the other (Figure 8B) (Gangwar et al., 2024b), similar to that observed in the putative apo state mentioned above (Figure 4C). The preserved LBD dimer, however, had the interface between monomers severely altered compared to the two-fold symmetrical LBDs in the apo closed state. Indeed, the D1–D1 interface of the preserved LBD dimer in this asymmetric structure was ruptured, resulting in a pronounced separation of the D1 lobes compared to the closed apo-state structures. In addition, the dimer lost its two-fold rotational symmetry, which characterizes the apo closed state. This loss of symmetry, evidenced by the emergence of a side cleft between protomers of the LBD dimer, is a hallmark of desensitization of AMPARs in complex with auxiliary subunit GSG1L which slows the recovery from desensitization substantially (Twomey et al., 2017b; Klykov et al., 2021).

One possible interpretation of the available agonist-bound non-conducting KAR structures in pseudo four-fold and asymmetric LBD conformation is that they represent different steps of the desensitization process, which starts similar to AMPARs, with the rupture of D1-D1 interfaces in two intact LBD dimers, followed by dissociation of one LBD dimer and then the second (Gangwar et al., 2024b). This hypothetical mechanism with deeper desensitization of KARs compared to AMPARs is consistent with slower recovery from desensitization observed in KARs compared to AMPARs (Gangwar et al., 2024b; Zhou et al., 2025).

In this mechanism, the asymmetric state reported recently (Figure 8B) (Gangwar et al., 2024b) is likely an “intermediate” step of KAR desensitization. While the initial, “weakly-desensitized” AMPAR-like state for wild-type KAR is currently not available, it is most likely similar to the state represented by the recent mutant GluK2 structure (K676C and N802C) in the agonist-bound non-conducting state trapped by disulfide crosslinking across the LBD dimer interfaces (Zhou et al., 2025) (Figure 8A). Overall, KAR desensitization appears to work as a multistep process, with a shallow desensitized state characterized by two-fold symmetry of the LBD layer, consecutively progressing through an asymmetric conformation to a deeply desensitized state with the four-fold symmetrical LBD layer and completely dissociated LBD dimers (Figures 8A–C). A puzzling question that remains to be answered: what drives KAR recovery from the “deep” desensitized state in this model? While it is conceivable that the forces generated by Glu-induced LBD closures drive ∼100° rotations of subunits B and D LBDs to enter the “deep” desensitized state, forces that push them to rotate back upon Glu dissociation, aside from diffusion, remain unclear. It is important therefore to point out that the “intermediate” and “deep” desensitized states could simply be artefacts of cryo-EM sample preparation.

### 4.3 Activated open conducting state

**FIGURE 9:**
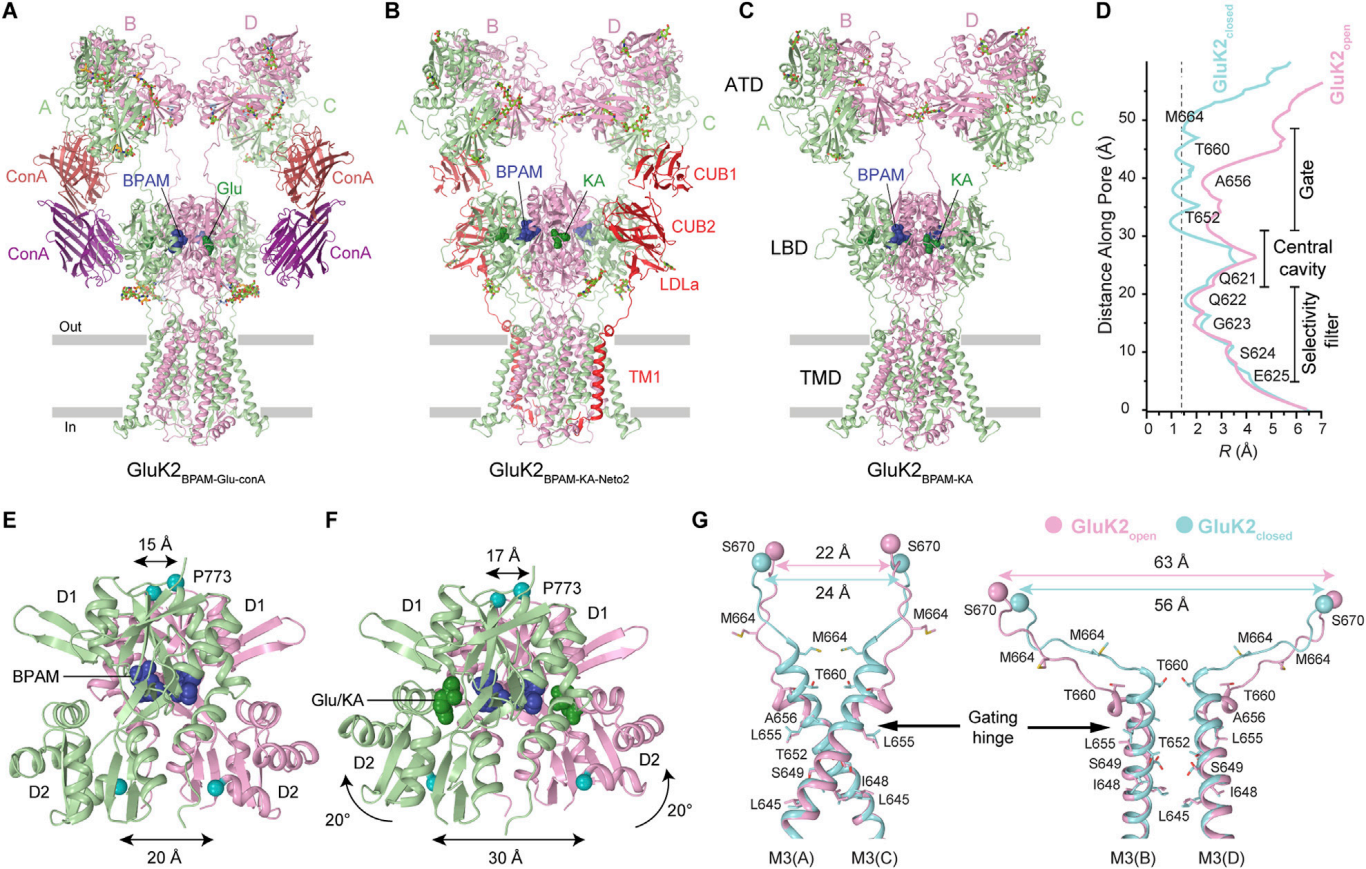
Agonist bound open-state and mechanism of KAR activation. **(A)** Open-state structure of GluK2 bound to positive allosteric modulator BPAM344 (BPAM), lectin Concanavalin A (ConA), and agonist glutamate (Glu) (PDB: 9B36). **(B)** Open-state structure of GluK2-Neto2 complex bound to BPAM and agonist kainic acid (KA) (PDB: 9N4T). **(C)** Open state structure of GluK2 alone bound to BPAM and KA (PDB: 9N4N). **(D)** Pore radius for the closed and open states. The vertical dashed line denotes the radius of a water molecule, 1.4 Å. **(E,F)** LBD dimers in the closed GluK2_BPAM_ **(E)** and open GluK2_Glu-ConA-BPAM_ **(F)** structures. Distances between Cα atoms of S670 and P773 are indicated. **(G)** Superposition of the pore-lining segments M2 and M3 and M3-S2 linkers in subunits A and C (left) and B and D (right), with residues lining the pore in the gate region shown as sticks. Distances between Cα atoms of S670 are indicated.

Recent studies have provided a significant breakthrough in understanding the activation gating mechanism in KARs. Due to the rapid kinetics of KAR activation, deactivation, and desensitization (<5 ms), capturing the receptor in its open state had proven to be challenging. This was addressed pharmacologically by identifying PAMs, including BPAM and the plant lectin Concanavalin A (ConA), which slowed down desensitization to the timescale of seconds (Gangwar et al., 2024b). Co-application of BPAM and ConA synergistically decelerated receptor desensitization, thereby extending the duration of activation. Subsequent application of Glu and plunge freezing within 3 s using time-resolved cryo-EM enabled the capture of the KAR open state (Figure 9A). The determined open-state structures demonstrated that ConA functions as a spacer between the ATD and LBD layers, while BPAM works as a stabilizer of the LBD dimer interface (Figures 9A–C) (Gangwar et al., 2024b). By comparing the closed- and open-state structures, it became possible to deduce the KAR activation mechanism. Upon Glu binding, LBD clamshells close around the agonist, leading to the separation of the D2 lobes within LBD dimers and an expansion of the LBD layer, marked by a pronounced increase in the distance between the D2 lobes of the diagonally arranged subunits B/D (Figures 9B–D). The LBD layer expansion generates increased tension in the LBD-TMD linkers, inducing kinking of all four M3 helices at a gating hinge around the residue L655 (Figure 9D).

KAR activation appears to be similar to that of AMPARs, likely proceeding via a pre-active state, in which Glu is bound but the ion channel remains closed (Gangwar et al., 2024b). However, the opening of the GluK2 channel involves conformational kinks of all four M3 helices at the gating hinge (Gangwar et al., 2024b). In contrast, the PAM cyclothiazide (CTZ)-bound open-state AMPARs exhibit kinking of M3 helices in subunits B and D only (Twomey et al., 2017a; Herguedas et al., 2019), while PAM-free open-state AMPARs at high temperature show slight bending of M3 helices in subunits A and C as well, although very modest compared to kinking of these helices in GluK2 (Gangwar et al., 2024b; Kumar Mondal et al., 2025). One possible explanation for this difference is that among various subconductance states of the channel (O1-O4), the Glu, ConA, and BPAM bound GluK2 represents the maximal conductance state (O4), while AMPAR structures represent lower (O1/O2) conductance states (Twomey et al., 2017a; Herguedas et al., 2019; Yelshanskaya et al., 2022). Alternatively, LBD–TMD linkers in AMPARs and KARs, with distinct amino acid sequences, may guide LBD layer expansion forces differently toward the M3 helix. This is supported by a ∼12° rotation of the LBD layer in open GluK2 *versus* GluA2 (Gangwar et al., 2024b). Additional studies using single-channel recordings, single-molecule FRET (smFRET), and cryo-EM are needed to confirm and structurally characterize the existence of KARs in multiple conductance states.

Several more open-state KAR structures were recently solved for homotetrameric GluK2 and the GluK2-Neto2 complex (Figures 9B–D) (Gangwar et al., 2025). In this investigation, kainic acid (KA), instead of Glu, was employed as a KAR agonist, along with BPAM. Utilizing time-resolved cryo-EM, the receptors were captured in the open-state conformations after incubation with BPAM and brief application of KA. The overall architecture of the receptor core for GluK2 alone or in complex with Neto2 closely resembled that of the GluK2-ConA-BPAM-Glu complex (Gangwar et al., 2024b). Despite the different agonists or binding partners, the activation mechanism and pore radius in all the open-state structures of GluK2, GluK2-ConA, and GluK2-Neto2 remain very similar (Figures 9A–G). Although Neto2 binding to the receptor does not appear to alter the behavior of individual or dimeric ATDs, LBDs, or the ion channel pore, the presence of Neto2 inhibited tightening of the interface between the two LBD dimers during activation and consequently decelerated the kinetics of receptor deactivation (Gangwar et al., 2025).

## 5 Future perspectives

Functional KARs are located at pre-, post-, and extra-synaptic sites, where they help to control neurotransmitter release, trigger postsynaptic depolarization, and regulate ion channels and cell excitability (Lerma, 2006; Vincent and Mulle, 2009). Furthermore, KARs are involved in various types of synaptic plasticity, including long-term potentiation (LTP) and long-term depression (LTD) (Sihra and Rodríguez-Moreno, 2013; Sihra et al., 2014). Consistent with their important physiological roles, KARs have been implicated in a number of neurological diseases, including epilepsy (Falcón-Moya1 et al., 2018), pain (Bhangoo and Swanson, 2013), ischemic brain injury (Du et al., 2009), anxiety/stress (Zhuo, 2017), ataxia and speech impairment (Stolz et al., 2021), schizophrenia, alcohol abuse disorder, bipolar disorder and depression, and intellectual disabilities (Lerma and Marques, 2013; Arora et al., 2018; Negrete-Díaz et al., 2022).

Due to their modulatory role in neuronal circuits, KARs represent promising therapeutic targets. However, the development of selective KAR agonists and antagonists has lagged behind the development of NMDA and AMPA receptor ligands, largely due to cross-reactivity. Therefore, many agonists and antagonists act on different iGluR types and highly specific KAR ligands are missing. Over the past years, numerous ligands (for example, NBQX, NS102, NS3763, LY382884, LY377770, GYKI53655, UBP296, UBP301) with varying potency and selectivity have been synthesized to modulate AMPARs and KARs, several of which exhibit neuroprotective properties and beneficial effects in preclinical models of stroke, myocardial infarction, and brain injury (Matute, 2010). One of these compounds, PMP, received an FDA approval for treatment of epilepsy, which acts on both AMPARs and KARs (Taniguchi et al., 2022; Chałupnik and Szymańska, 2023). Despite these advances, further work is required to evaluate the potential toxicity, optimize dosing regimens, refine molecular structures, and assess pharmacokinetic properties, including bioavailability of the ligands modulating KAR functions.

Recent progress in cryo-EM has elucidated critical conformational states of KARs, demonstrating how PAMs and auxiliary protein Neto2 stabilize receptor conformations by modulating interactions at the ATD and LBD dimer interface. Despite rapid progress over the last decade across physiological, pharmacological, and structural fields, several pivotal questions remain unresolved. For instance, investigations aiming to characterize the complete gating cycle of KARs encompassing transient states between resting, open, and desensitized states at near physiological conditions are integral to understand functional behavior of KARs in neurotransmission. A particularly critical area involves elucidating the structural impacts of *de novo* disease mutations in KARs, which will delineate molecular mechanisms of KAR-related neuropathies. Moreover, the structural elucidation of the different splice variants and those involving the Q/R site in the M2 pore loop will be instrumental for understanding their role in receptor trafficking and Ca^2+^ permeability. Advances in membrane protein engineering, protein biochemistry techniques (i.e. nanodisc reconstitution), and improved cryo-EM sample preparation are expected to address current barriers in resolving KARs structures and capturing their transient intermediates. Concurrently, drug development efforts will benefit from atomic-resolution insights into competitive antagonism, allosteric modulation, and ion channel block. As the field advances, multidisciplinary efforts linking high-resolution structural data to functional genomics and disease models will be crucial to unlocking the full therapeutic potential of KARs, thereby addressing significant unmet medical needs in treatment of various neuropsychiatric, neurological, and neurodegenerative diseases.
